# Anti-psychotic prescription pattern: A preliminary survey of Psychiatrists in India

**DOI:** 10.4103/0019-5545.70982

**Published:** 2010

**Authors:** Sandeep Grover, Ajit Avasthi

**Affiliations:** Department of Psychiatry, Postgraduate Institute of Medical Education and Research, Chandigarh - 160 012, India

**Keywords:** Anti-psychotic, India, prescription patterns

## Abstract

Although anti-psychotic medications are available in India since a long time, little is known about the prescription patterns of Indian psychiatrists. An email survey was sent to 1100 psychiatrists, of which 168 responded. The three most commonly prescribed anti-psychotics were risperidone, olanzapine, and haloperidol. It was also found that typical anti-psychotics comprise of 25.15% (SD=21.66; range 0–100) of all prescriptions and in about 22.36% of the cases the psychiatrists were using more than one anti-psychotic in the same patient.

## INTRODUCTION

Anti-psychotic medications have been available in India since a long time. Over the years the anti-psychotic pharmacopenia has broadened and almost all the new anti-psychotics are available in India. Based on the side-effect profile over the years, atypical anti-psychotics have become the preferred choice and most of the recent guidelines also recommend the use of atypical agents as the first line of treatment for schizophrenia.[1,2] However, recent evidence shows that there is risk of developing a metabolic syndrome with some of the atypical anti-psychotics.[3] Based on this evidence, a joint declaration by the American Psychiatric Association and American Diabetic Association has formulated the monitoring guidelines when using atypical anti-psychotics and they have also provided a guideline as to the relative risk of the metabolic syndrome with various atypical anti-psychotics.[4]

There are very few studies that have evaluated the anti-psychotic prescription patterns from India. In one of the early studies, Khanna *et al*.[5] evaluated the psychotropic drug prescription pattern in chronic long-stay patients at Ranchi and compared the prescription trends in 1984 and 1988. Most of the patients whose prescriptions were reviewed were suffering from schizophrenia. More than one anti-psychotic medication was prescribed to 13% of cases in 1984, which fell down to 7% in 1988. Moreover, it was seen that very few patients received anticholinergic agents and the use of benzodiazepines increased over the years (4% in 1984 and 10% in 1988), which the authors attributed to the development of distressing tardive dyskinesia over the years.[5] Padmini Devi *et al*.[6] evaluated the discharge prescription of all patients of schizophrenia from St. Johns Medical College, Bangalore, during the period January 2003 to December 2003. They reported that risperidone was the most commonly prescribed anti-psychotic (56.17%), followed by olanzapine (21.34%), and quetiapine (3.93%). Typical anti-psychotics were used only in 15.73% of the cases and polypharmacy (concurrent use of more than one anti-psychotic) was seen in 9% of the cases.[6] In another study from Jammu, Shanwey *et al*.[7] evaluated the prescription of 270 outpatients, during the period 1 January to 30 June, 2004, and reported that a fixed dose formulation of trifluperazine, chlorpromazine, and trihexyphenidyl (parkinforte) was used in 45.4% of the cases followed by chlorpromazine [36.3%] and quetiapine [34.5%]. The authors also found that typical anti-psychotics were used in 82.72% of the cases and polypharmacy was seen in 72.72% of the cases.[7] In a recent study, Trivedi *et al*.[8] from Lucknow evaluated the prescription of 100 patients attending the Psychiatry Outpatient Clinic. They reported that olanzapine was the most commonly used anti-psychotic medication (64%) and it was followed by risperidone (48%) and Inj flupenthixol (17%). Typical anti-psychotics were used in 41% of the cases and polypharmacy was seen in 55% of the cases. The anti-psychotics for which a chlorpromazine equivalent dose was available – it was seen that a majority of the cases (61.83%) received between 100 to 400 mg of chlorpromazine equivalent doses of various anti-psychotics and only a few cases (13.14%) received more than 600 mg of the chlorpromazine equivalent dose.[8]

From the above-mentioned data it is clear that whatever data is available has come from the public sector hospitals and represents the prescription pattern specific to that center, and it is probably influenced by medications available in the dispensary of the hospital.

In this background, we carried out an e-mail survey of psychiatrists practicing in India – about their prescription pattern in psychotic patients.

## MATERIALS AND METHODS

For this study a small questionnaire consisting of three questions was designed to get the basic information about the prescription of various anti-psychotics in psychotic patients. The three questions asked were as follows:

1. Name three anti-psychotics most commonly prescribed by you and their average dose.
2. In what percentage of cases do you prescribe typical anti-psychotics?
3. In what percentage of cases do you concurrently prescribe more than one anti-psychotic?

This questionnaire was sent to 1100 psychiatrists by one of the authors (AA), in the month of December 2008. The psychiatrists were informed that the data would be used for professional purposes. The questionnaire was sent one to three times, varying on the response, and in total 175 responses were received, of which seven responses were from Indian psychiatrists practicing outside India. As our aim was to study the prescription pattern of psychiatrists practicing in India, these seven responses were not included in the final analysis. If any of the psychiatrists gave a range (for example 20–40%) to a particular question, the middle value of the range (30% in this example) was taken for the final analysis. The data obtained in relation to the three most commonly prescribed anti-psychotics was pooled together to obtain the composite figure of commonly prescribed anti-psychotics. The data was analyzed using SPSS-14.

## RESULTS

Of the 168 psychiatrists who responded, three gave the name of only one anti-psychotic and two named only two anti-psychotics, instead of the required three most commonly prescribed anti-psychotics (question no-1 of the survey). The three most commonly prescribed anti-psychotics were risperidone, olanzapine, and haloperidol. Of the 168 psychiatrists who responded to the survey, two of the three preferences of 88.69% of the psychiatrists were risperidone and olanzapine. Haloperidol was one of the three choices of 32.14% of the psychiatrists. Quetiapine was one of the three choices of 28.57% of psychiatrists.

Further it was found that 45.7% of the psychiatrists reported at least one typical anti-psychotic among the three most commonly prescribed anti-psychotics.

When all the three preferences were pooled as shown in Table 1, it was seen that risperidone and olanzapine comprised 30% each of the prescriptions. Haloperidol accounted for 10.9% of the prescriptions, followed by quetiapine (9.7%).

**Table 1:** Commonly prescribed anti-psychotics with the mean dosages

Anti-psychotics	(N=496)	%	Mean dose in mg	Range in mg
Risperidone	149	30.0	4.90± 1.44	1–12
Olanzapine	149	30.0	14.22± 4.96	2.5–40
Haloperidol	54	10.9	11.82± 6.81	3–40
Quetiapine	48	9.7	365.11± 147.98	50–750
Trifluperazine	29	5.8	13.10± 4.69	5–25
Aripiprazole	20	4.0	20.29± 5.98	10–40
Amisulpride	19	3.8	337.50± 170.90	50–800
Clozapine	11	2.2	227.27± 130.6	50–400
Chlorpromazine	7	1.4	246.42± 196.54	75–600
Flupenthixol	4	0.8	23.33± 5.77	20–30
Ziprasidone	3	0.6	90± 42.42	60–120
Loxapine	1	0.2	100	
Zuclopenthixol	1	0.2	100	
Levosulpiride	1	0.2	200	

The mean dose of various anti-psychotics along with the range is shown in Table 1.

The survey also found that typical anti-psychotics constituted 25.15% (SD=21.66; range 0–100) of all prescriptions (question No. 2 of the survey) and in about 22.36% (SD=22.50; range 0–100) of the cases the psychiatrists used more than one anti-psychotic in the same patient (question No. 3 of the survey).

A significant positive correlation was seen between the percentage use of typical anti-psychotics and use of more than one anti-psychotic (Pearson product moment correlation coefficient=520***).

## DISCUSSION

There is a lack of national level data as to the prescription patterns of various psychotropic medications in India. This survey was a small effort to generate a national level data to understand the prescription patterns of psychiatrists practicing in India. As there is no national level data to compare, we will discuss the findings of this survey in the light of available data from the west and the implications of the findings of this survey.

This survey demonstrates that risperidone and olanzapine are the two most commonly prescribed anti-psychotics by the responding psychiatrists. Both the medications being two of the three choices of about 88.69% of psychiatrists suggests that these medications form at least two-thirds of all the prescriptions of anti-psychotics in India. Haloperidol was the third most commonly preferred anti-psychotic. Findings of our survey are in line with the findings of recent prescription surveys of Padmini Devi *et al*.[6] and Trivedi *et al*.[8], who have also reported that olanzapine and risperidone are the two most commonly prescribed anti-psychotics. However, our survey does not support the findings of Shanwey *et al*.[7] who reported that typical anti-psychotics are used more frequently than atypical anti-psychotics.

Our survey suggests that most of the psychiatrists are comfortable with using the atypical anti-psychotic in most of their patients. Moreover, the prescription pattern is possibly influenced by the available guidelines and literature, which suggests that these medications are efficacious in both positive and negative symptoms. However, when we look at the risk of the metabolic syndrome, as suggested by the joint statement of the American Psychiatric Association and the American Diabetic Association, the findings of this survey are alarming. Olanzapine has been rated as the agent, next only to clozapine, which leads to the metabolic syndrome.[4] Similarly, the risk with risperidone is also high, but slightly less compared to olanzapine and clozapine.[4] Hence, our findings suggest that with the current prescription pattern, patients are at a high risk of developing the metabolic syndrome.

This survey also reflects that the use of typical anti-psychotics is waning as compared to atypical anti-psychotics and this is in concordance with the observation made in a survey on practice patterns and treatment choices among psychiatrists in New Delhi, wherein, it has been observed that very few patients are prescribed typical anti-psychotics.[9] Furthermore, our survey also provides credence to the observation made by Varghese,[10] who pointed out that among the recently passed postgraduate psychiatrists there are few who are comfortable with using typical anti-psychotics.

In this survey we found that in nearly one-fourth of the patients (22.36%), the psychiatrists were using more than one anti-psychotic in the same patient. This figure is about half, as found in the actual survey of prescriptions done in the East Asian countries.[11] It is similar in this case when compared with the report of Trivedi *et al*.[8] from Lucknow, but much lower than that reported by Shanwey *et al*.[7] However, it is more than that reported by Padmini Devi *et al*.[6] and Khanna *et al*.[5] This large variance in polypharmacy probably reflects not only individual differences, but also differences in the prescription patterns among various centers in the country.

Limitations and conclusion

It is important to consider the limitations of this survey. It included the opinions of only 168 psychiatrists. Considering that there are about 3500 psychiatrists in India, this survey does not reflect the true prescription pattern of all the psychiatrists in India. In future, a larger survey should be conducted covering many areas of the prescription pattern, such as, prescription in specific situations, prescription in special population, switching strategies, monitoring strategies for side effects, relationship between the type of medication prescribed and the psychopathology, external factors leading to the selection of specific medications, and so on. There is a need to collect national level data of the actual prescriptions handed over to the patients in order to have a better understanding of changes, if any, in the prescription patterns.
